# Oxidative stress and epigenetics in ocular vascular aging: an updated review

**DOI:** 10.1186/s10020-023-00624-7

**Published:** 2023-02-27

**Authors:** Bowen Zhao, Lijia Zhu, Meng Ye, Xiaotong Lou, Qianxue Mou, Yuanyuan Hu, Hong Zhang, Yin Zhao

**Affiliations:** Department of Ophthalmology, Tongji Hospital, Tongji Medical College, Huazhong University of Science and Technology, Wuhan, 430030 China

**Keywords:** Vascular aging, Microvascular dysfunction, Oxidative stress, Epigenetics, Diabetic retinopathy, Age-related macular degeneration

## Abstract

Vascular aging is an inevitable process with advancing age, which plays a crucial role in the pathogenesis of cardiovascular and microvascular diseases. Diabetic retinopathy (DR) and age-related macular degeneration (AMD), characterized by microvascular dysfunction, are the common causes of irreversible blindness worldwide, however there is still a lack of effective therapeutic strategies for rescuing the visual function. In order to develop novel treatments, it is essential to illuminate the pathological mechanisms underlying the vascular aging during DR and AMD progression. In this review, we have summarized the recent discoveries of the effects of oxidative stress and epigenetics on microvascular degeneration, which could provide potential therapeutic targets for DR and AMD.

## Background

Vascular aging is an irreversible pathophysiological process involving the whole body of the elder. It is regarded as the common character in age-related cardiovascular and microvascular diseases, such as atherosclerosis, hypertension, cerebrovascular pathologies and retinal vascular diseases (Ungvari et al. 2020). The aging population is always growing, and the vascular disorders threaten seriously the public health. Therefore, it is an urgent task to elucidate the mechanisms underlying the vascular aging, in order to explore therapeutic strategy to attenuate the progression of relevant diseases.

Retinal vascular diseases rank among the leading causes of blindness worldwide, in which diabetic retinopathy (DR) is most prevalent with a large afflicted population (Gahlaut et al. 2015). The outer retina is nourished by choroidal vasculature, and choroidal ischemia damages both choroidal and retinal structure which causes visual loss during the process of age-related macular degeneration (AMD). Vascular aging plays a crucial role in the pathogenesis of these diseases, manifested as vascular cellular degeneration, blood-retinal barrier (BRB) breakdown and pathological neovascularization (NV). As broadly reviewed, there are numerous pathophysiological mechanisms of vascular aging, including oxidative stress, inflammation, genomic instability, cellular senescence and epigenetic alterations (Ungvari et al. 2018; Jia et al. 2019; Paneni et al. 2017). The present review focuses on the involvement of oxidative stress and epigenetic dysregulation in vascular aging during DR and AMD.

## Oxidative stress and vascular aging

The retina is a highly metabolically active tissue with high oxygen consumption (Corso-Diaz et al. 2018). Oxygen metabolism produces reactive oxygen species (ROS), which can induce cellular damage by the excessive accumulation in pathological conditions. The imbalance between production and elimination of ROS refers to oxidative stress (Pizzino et al. 2017). Especially high oxygen consumption makes the retina susceptible to oxidative stress (Jarrett and Boulton 2012). In retinal vascular diseases, oxidative stress is implicated from the onset, and coordinates with ischemia, vascular inflammation and angiogenesis during the progression of visual impairment (Dammak et al. 2021).

### Association of vascular aging with oxidative stress in DR

DR is one of the severe complications of diabetes mellitus, and aging and hyperglycemia are listed as the leading risk factors of DR. The metabolic abnormalities induce dysregulation of mitochondrial biogenesis and ROS overproduction in retinal vessels (Giacco and Brownlee 2010; Ungvari et al. 2008; Ihnat et al. 2007), which are responsible for vascular aging. There are many signs of vascular aging in DR progression, (1) endothelial degeneration; (2) pericyte loss; (3) cellular responses to ischemia damage at both early preclinical stage of the disease and advanced stage with appearance of proliferative DR (Stitt et al. 2016).

#### Endothelial degeneration

In human retina samples suffered from non-proliferative DR, numerous microaneurysms were detected adjacent to arterioles in retinal vasculature. Moreover, the microvascular abnormalities were also determined by the absence of smooth muscle covering in pre-capillary arterioles and the increment of non-perfused acellular capillaries (Stitt et al. 2016; Gardiner et al. 2007). Acellular capillaries are characterized by loss of endothelial cells, resulting ultimately in the degeneration or obliteration of capillaries (Gardiner et al. 2007; Armulik et al. 2005). In STZ-induced diabetic mice, the number of acellular capillaries notably increased in retinal vasculature, accompanied with vascular hyperpermeability (Tang et al. 2022; Shan et al. 2017). Oxidative stress caused apoptosis of endothelial cells with the appearance of BRB breakdown in DR (Tang et al. 2022). Endothelial apoptosis was attributable to activation of NADPH oxidase, accumulation of ROS and mitochondrial dysfunction induced by high glucose (HG) (Tang et al. 2022; Quagliaro et al. 2003). Moreover, diabetic retinas suffered from depressed activity of superoxide dismutase (SOD), an antioxidant enzyme (Kowluru et al. 2006). Accordingly, SOD overexpression improved antioxidative potential and consequently ameliorated capillary degeneration observed in DR (Kanwar et al. 2007). Microglia phagocytosis-induced endothelial loss was also responsible for the formation of acellular capillaries in an experimental DR model. Inhibition of phagocytic activity and excessive activation of microglia could prevent endothelial loss and BRB disruption through repressing inflammation and oxidative stress (Xie et al. 2021; Zhang et al. 2019).

#### Pericyte loss

Besides endothelial cells, pericytes are another primary cellular constituent in the retinal microvasculature, which play a vital role in regulating blood flow in capillaries as well as maintaining the integrity of the BRB (Attwell et al. 2016). Pericyte loss is an early phenotype of microvascular aging in DR (Beltramo and Porta 2013; Cacicedo et al. 2005), prior to the endothelial loss (Stitt et al. 2016; Beltramo and Porta 2013). In retinas of diabetic mice, retinal vascular leakage was detected, as a consequence of pericyte loss (Yun et al. 2018; Jiang et al. 2020). HG treatment induced apoptosis of human retinal pericytes in response to oxidative damage, which was recognized by the decreased activities of catalase and SOD, and the elevated malondialdehyde concentration (Zeng et al. 2022). Pericyte apoptosis arisen from hyperglycemia could be alleviated through regulating Nrf2-mediated antioxidative responses (Zeng et al. 2022). In an in vitro experiment simulating early diabetes, apoptosis of cultured primary retinal pericytes was attenuated by NADPH oxidase inactivation or overexpression of antioxidant genes (Cacicedo et al. 2005; Chen et al. 2006). The interaction of pericytes with endothelial cells is indispensable for microvascular stabilization, while it is disrupted due to vascular aging in DR (Armulik et al. 2005; Huang 2020). Hyperglycemia disrupted the adherence of pericytes to endothelium, which was manifested as increased migrating pericytes (Pfister et al. 2008). In 3-month *Ins2*^*Akita*^ diabetic mice, before the appearance of pericyte loss, migrating or extravascular pericytes were already visible in retinal capillaries, which indicated the depressed interaction between endothelial cells and pericytes (Hu et al. 2017). In addition, the apoptosis of human retinal pericytes induced by HG treatment was aggravated by incubation with extracellular matrix produced by HG-conditioned endothelial cells. Then, administration of a NADPH oxidase inhibitor reversed the pericyte loss (Beltramo et al. 2009; Ahmed et al. 2020). Overall, oxidative stress serves as a crucial pathological mechanism in diabetic injuries to endothelial cells and pericytes, and therefore antioxidative therapies can rescue microvessels and BRB function.

#### Cellular responses to ischemia

An early functional change of vascular aging in diabetic retinas is the depressed blood flow with dysregulation (Stitt et al. 2016). Preclinical vascular abnormalities were detected in ophthalmic arteries of diabetes patients, identified as aberrant flow velocity waveforms and impaired autoregulatory response of the vessel diameter to inhaled O_2_ (Lockhart et al. 2014). Microvascular blood flow is mainly controlled by resistance arterioles, in which endothelium participates in vasomotor regulation (Hein et al. 2020). Travis W. Hein reported the impairment of endothelium-dependent dilation mediated by nitric oxide (NO) synthase in retinal arterioles at the early stage of experimental diabetes (Hein et al. 2020, 2016). During vascular aging, oxidative stress arisen from increased expression of nuclear factor-κB (NF-κB) p65 and NADPH oxidase in endothelium (Donato et al. 2007) causes declined NO levels, enhanced NO synthase activity and impaired bioavailability of NO, all of which contribute to compromised endothelium-dependent dilation (Ungvari et al. 2018; Loo et al. 2000). Concomitant with disabled vasodilation, retinal blood flow decreased in both experimental diabetes animals (Geraldes et al. 2009; Mills et al. 2021) and diabetic patients (Safi et al. 2018). Besides depressed blood flow, vascular aging in response to diabetes manifests as thickening of basement membrane, insufficient oxygen delivery, increased plasma viscosity and microvascular degeneration, leading to ischemia and hypoxia in retina tissue (Kaur et al. 2008). Ischemia and hypoxia promote excessive production of vascular endothelial growth factor (VEGF) and trigger retinal NV as a consequence (Campochiaro 2015; Osborne et al. 2004). As NV increases, proliferative DR progresses with neovascular complications including vitreous hemorrhage, retinal detachment and neovascular glaucoma, which have detrimental effects upon vision (Antonetti et al. 2021). Oxidative stress is implicated in the process of angiogenesis (Kim and Byzova 2014). NV was detected in oxygen-induced retinopathy model with increased expression of VEGF, NADPH oxidase and superoxide formation (Wang et al. 2019). Inhibition of NADPH oxidase suppressed VEGF overexpression as well as retinal NV induced by ischemia and hypoxia (Al-Shabrawey et al. 2005). Lipid peroxidation is also related to VEGF expression and BRB breakdown in diabetic model (Ayalasomayajula and Kompella 2003; SanGiovanni and Chew 2005; Rodriguez et al. 2021). Excess ROS derived from NADPH oxidase and mitochondrial dysfunction contributes to VEGF expression and NV in DR (Deliyanti et al. 2020; Fukai and Ushio-Fukai 2020). Furthermore, in turn, VEGF stimulates ROS generation in endothelial cells and potentiates angiogenesis in a self-reinforcing manner (Fukai and Ushio-Fukai 2020; Colavitti et al. 2002).

**Fig. 1:**
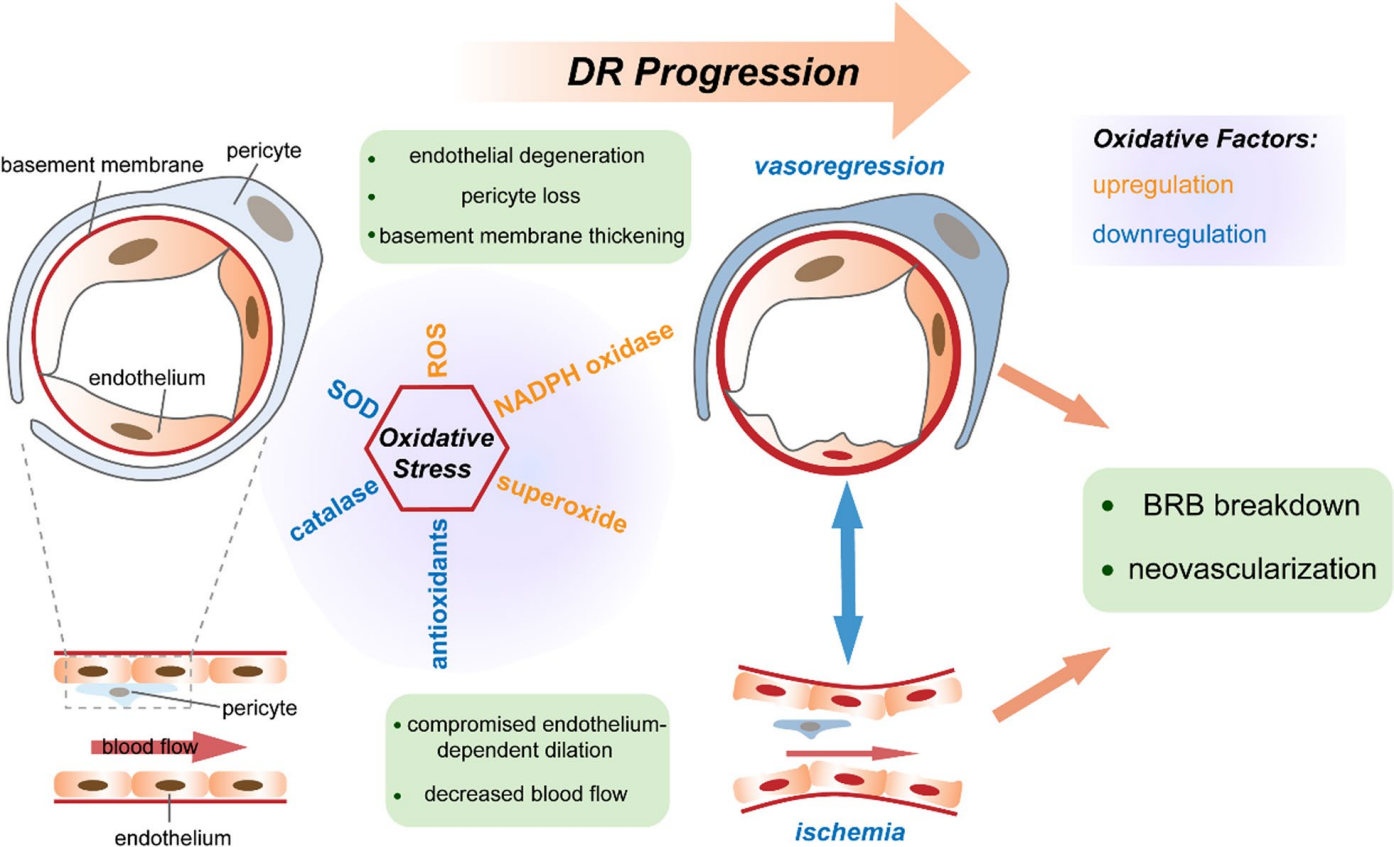
Schematic illustration of retinal vascular aging in response to oxidative stress in diabetic retinopathy (DR). Oxidative stress is manifested as increased levels of ROS, NADPH oxidase and superoxide, in contrast with decreased expression of SOD, catalase and antioxidants. Oxidative stress induces endothelial degeneration, pericyte loss and basement membrane thickening, which aggravates vasoregression in the process of DR. Concurrently, retinal ischemia occurred due to the compromised endothelium-dependent dilation and decreased blood flow in retinal microvessels. Vasoregression and ischemia coordinate with each other and eventually result in BRB breakdown and neovascularization during DR progression. ROS: reactive oxygen species, SOD: superoxide dismutase

As DR progresses, vasoregression and ischemia coordinate with each other to aggravate the BRB breakdown and retinal NV (Fig. 1). Vascular leakage induces the accumulation of extracellular fluid and instigates chronic inflammation. When the macula is invaded and loses the normally compact structure, diabetic macular edema (DME) develops (Antonetti et al. 2021). DME accelerates retinal neuronal abnormalities and seriously damages the visual acuity of DR patients, even with the danger of blindness (Gahlaut et al. 2015; Stitt et al. 2016; Antonetti et al. 2021).

### Relationship between vascular aging and oxidative stress in AMD

AMD is a progressive degenerative disease affecting the macular region of the retina, and threatens severely the visual function in aged people (Mitchell et al. 2018). According to the manifestation, AMD can be classified into early- or intermediate-stage AMD, and late-stage AMD. Early- or intermediate-stage AMD is characterized by the deposit of drusen in the subretinal space. Late-stage AMD includes two categories called neovascular (wet or exudative) AMD and atrophic (dry) AMD (Fleckenstein et al. 2021). Both advanced AMD can concomitantly occur and exacerbate the vision impairment synergistically (Kaszubski et al. 2016; Tisi et al. 2021). There are many risk factors identified for AMD, such as aging, smoking, hypertension, diabetes, and genetic predisposition. Among these, aging serves as the strongest pathogenic factor of AMD, proved by the increased morbidity and blindness-incidence with advancing age (Mitchell et al. 2018; Tisi et al. 2021; Joachim et al. 2015; Colijn et al. 2017; Thomas et al. 2021). Microvascular degeneration is the phenomenon of vascular aging in microcirculation (Ungvari et al. 2018). Multiple evidences have demonstrated the relationship between microvascular degeneration and AMD pathogenesis (Mullins et al. 2011a; Whitmore et al. 2015; Nassisi et al. 2021; Nesper et al. 2021; Biesemeier et al. 2014; Lee et al. 2018). In patients suffering from early AMD, vascular density of choriocapillaris decreased, and showed inverse association with subretinal drusen density (Mullins et al. 2011a). The number of ghost vessels within choriocapillaris was also correlated with drusen density in AMD macular sections, indicating the appearance of capillary loss (Whitmore et al. 2015). Optical coherence tomography angiography images of early and intermediate AMD illustrated blood flow deficits in choriocapillaris, which were responsible for the impairment of macular visual function (Nassisi et al. 2021). Ischemia attributable to depressed blood supply promotes VEGF production and initiates choroidal neovascularization (CNV) (Campochiaro 2000, 2015; Feigl 2007). In neovascular AMD, choriocapillaris flow deficits had impact on vascular complexity of CNV (Nesper et al. 2021). It was reported that loss of choriocapillaris occurred prior to the atrophy of retinal pigment epithelium (RPE), and aggravated RPE degeneration in geographic atrophy (Biesemeier et al. 2014). Other evidences confirmed that RPE atrophy preceded choriocapillaris loss in a feedforward loop (Seddon et al. 2016). Conversely, improved RPE organization was concurrent with the maintenance of choroidal vasculature (Yang et al. 2020). Furthermore, retinal capillary plexus of AMD patients showed lower vascular density compared to the controls, suggesting the involvement of retinal vasculature in AMD progression (Lee et al. 2018).

#### Neovascularization

Oxidative stress is listed as the major pathogenic mechanism of aging, which induces microvascular degeneration in the process of AMD (Ruan et al. 2021). Nuclear factor-erythroid 2-related factor-2 (Nrf2) is a key transcription factor that regulates the cellular defense against oxidative stress (He et al. 2020). Nrf2 deletion increased ROS generation and inhibited the expression of antioxidant enzymes (Hyeon et al. 2013). Retinas isolated from AMD donor eyes demonstrated a decrease in Nrf2 mRNA levels (Aberami et al. 2019). To investigate the role of Nrf2 in AMD pathogenesis, Zhao Z et al. performed genetic manipulation in mice and monitored ocular abnormalities during aging. Aged Nrf2-knockout mice developed subretinal drusen-like deposits, RPE degeneration and CNV, all of which resembled the features of human AMD (Zhao et al. 2011). Both cellular and animal experiments corroborated that RPE loss and CNV lesions were alleviated dependent on the upregulation of Nrf2 and its targeted antioxidant enzyme HO-1 (Yoshinaga et al. 2011). Pharmaceutical activation of Nrf2 suppressed VEGF-induced migration of retinal endothelial cells, and reduced the vascular hyperpermeability in a primate CNV model (Nakamura et al. 2019). Similarly, through scavenging ROS, administration of NADPH oxidase inhibitor attenuated CNV lesions. Moreover, ROS reduction was correlated with improved RPE barrier as shown by enhanced expression of junction proteins (Li et al. 2018). However, deletion of SOD1 (an antioxidant enzyme) contributed to CNV as well as RPE dysfunction during aging. Accordingly, SOD1-deficient mice could be used as AMD models to investigate the effect of oxidative stress on drusen formation, Bruch’s membrane thickening, loss of RPE integrity and pathological angiogenesis (Imamura et al. 2006). The antioxidative effect of resveratrol on vasculature has been widely reviewed (Xia et al. 2017; Li et al. 2019; Parsamanesh et al. 2021). On one hand, resveratrol counteracts NADPH oxidase-mediated production of ROS and mitochondrial superoxide generation to maintain cardiovascular function (Xia et al. 2017). On the other hand, resveratrol regulates the expression of antioxidants such as Nrf2 and histone deacetylase sirtuin 1 (Sirt1), that can relieve endothelium from oxidative stress (Parsamanesh et al. 2021). As reported, administration of resveratrol suppressed NF-κB activation in Choroid-RPE complex after laser photocoagulation, and prevented CNV via downregulation of VEGF (Nagai et al. 2014). In an in vitro AMD model, resveratrol attenuated hypoxia-induced proliferation of choroidal vascular endothelial cells through reducing VEGF release and activation of stress-activated protein kinases (Balaiya et al. 2013). In addition, RPE degeneration exacerbates the vascular impairment in response to oxidative stress in AMD. RPE challenged with oxidative damages secreted fibroblast growth factor, which promoted the proliferation of choroid endothelial cells (Eichler et al. 2008). In AMD, activated choroid endothelial cells migrate through Bruch’s membrane toward RPE and then invade the neurosensory retina. Mounting evidences have validated that RPE barrier dysfunction is a prerequisite for CNV breakthrough (Li et al. 2018; Ramshekar et al. 2021; Gehrs et al. 2006). In a laser-induced rat CNV model, inhibition of ROS generation in RPE counteracted VEGF production and CNV lesions (Li et al. 2018). Another study found that the area of CNV decreased after interfering the expression of a subunit of NADPH oxidase complex in RPE (Li et al. 2008).

#### Immune responses

Accumulation of oxidative stress during aging damages Choroid-RPE complex, and thereafter immune responses are initiated (Fleckenstein et al. 2021). In a retinal degeneration model, increased deposition of complement factors appeared in subretinal space after exposure to oxidative damage. Inhibition of innate immune system by Toll-like receptor (TLR) 2-deletion protected against complement accumulation and mononuclear cell infiltration, and therefore improved RPE survival in stressed retinas (Mulfaul et al. 2020). Genetic polymorphisms in the complement factor H (CFH) gene elevate the risk of the onset and progression of AMD (Lipecz et al. 2019). CFH is the main negative regulator of alternative pathway in complement system, while CFH variants elicit uncontrolled activation of alternative pathway that induces excessive signal amplification and downstream events such as membrane attack complex (MAC) formation (Lipecz et al. 2019; Mullins et al. 2011b). Growing evidences have shown the accumulation of MAC in aging choriocapillaris especially in those suffered from AMD (Chirco et al. 2016, 2017; Mullins et al. 2014). The abundance of MAC increases with advancing age, and especially it is much higher in Choroid-RPE of AMD patients than that of aged people without AMD (Mullins et al. 2014). Besides choriocapillaris and Bruch’s membrane, MAC can also be detected in drusen deposits and choroidal neovascular membrane (Mullins et al. 2014; Anderson et al. 2002). In a murine CNV model with MAC accumulation, anti-complement treatment prevented MAC deposition and CNV formation, concomitant with the downregulation of angiogenetic factors (Bora et al. 2005). Oxidative stress accentuates the cellular susceptibility to MAC injury. RPE death induced by MAC was aggravated by either acute sublethal H_2_O_2_ or chronic low-dose H_2_O_2_ challenge (Yang et al. 2014). In response to age-related oxidative insults, complement system is activated to generate a series of pro-inflammation responses. As the terminal event of the activated complement cascades, MAC formation regulates cell lysis, release of chemokines to recruit inflammation cells, and hyperpermeability of capillaries (Whitmore et al. 2015; Lipecz et al. 2019; Mullins et al. 2014; Cabrera et al. 2016). It was reported that activation of alternative complement pathway induced MAC deposition on the surface of choroidal endothelial cells followed by cell lysis. To mimic vascular aging in AMD, choroidal endothelial cells were cultured to high passages to establish replicative senescence. The study revealed that senescent cells underwent a greater extent of cell lysis as a result of MAC deposition, indicating the synergistic effect of senescence and complement injury (Cabrera et al. 2016). Senescence is the primary process of aging, which is concomitant with oxidative stress and chronic inflammation in many age-related pathologies including AMD (Tchkonia et al. 2013). Senescence-associated secretory phenotype (SASP) denotes that senescent cells secrete plenty of proinflammatory cytokines, chemokines and proteases. Through SASP, senescence cells attract immune cells to modify focal microenvironment (Tchkonia et al. 2013; Chen and Xu 2015). It has been thoroughly and extensively reviewed that activated microglia migrates from inner retina into damaged Choroid-RPE with a morphological and functional transformation in the process of AMD (Karlstetter et al. 2015; Langmann 2007; Ardeljan and Chan 2013), so that the activation of microglia would not be discussed here again. Another subset of resident immune cells is identified as perivascular macrophages (Chen and Xu 2015). During aging, increased density of choroidal macrophage was detected concurrent with progressive choroidal thinning and vascular atrophy (Yang et al. 2020). Senescent cells secrete excessive ROS, which can augment SASP propagation (Blasiak 2020). In a murine CNV model, eliminating ROS by resveratrol reduced inflammatory factors and macrophage infiltration, and then suppressed CNV development. The study implied the detrimental effect of macrophages on choroidal injuries during AMD (Nagai et al. 2014). Macrophage recruitment is directed by chemokines such as chemokine (C-C motif) ligand 2 (CCL2, also known as MCP-1). Increased CCL2 secretion was observed in Choroid-RPE complex of aged mice. Genetic deletion of CCL2 or its cognate receptor CCR2 suppressed macrophage recruitment to senescent tissues. Intriguingly, both genotypes developed drusen deposits, Bruch’s membrane thickening, RPE degeneration and CNV invasion with advancing age, which recapitulate the AMD features. In addition, aged CCL2 or CCR2 knockout mice suffered from complement activation and immune complex deposition in choroidal vessels. The explanation of these phenotypes was that inactivation of chemokines abrogated macrophage recruitment to the lesions, and consequently depressed the clearance of focal complement deposits due to the absence of macrophages (Ambati et al. 2003). The results revealed the indispensable role of macrophage in maintaining inflammatory homeostasis during AMD. Consistently, another investigation also validated the contribution of macrophages to relieving AMD impairments. Depletion of macrophages led to progressive choroidal vascular atrophy and RPE dysfunction. When the depletion was halted, macrophages regenerated spontaneously, followed by an arrest in the degenerative alterations (Yang et al. 2020). It is concluded that choroidal resident macrophages are devoted to the clearance of perivascular insults to maintain homeostasis under normal conditions. However, pathological dysregulation of inflammatory cascades instigates overactivation of macrophages, which causes an imbalance between the tissue impairment and repairment. Accordingly, either overaction or disability of endogenous macrophages is responsible for choroidal degeneration and retinal injuries in AMD (Yang et al. 2020; Raoul et al. 2010).

**Fig. 2:**
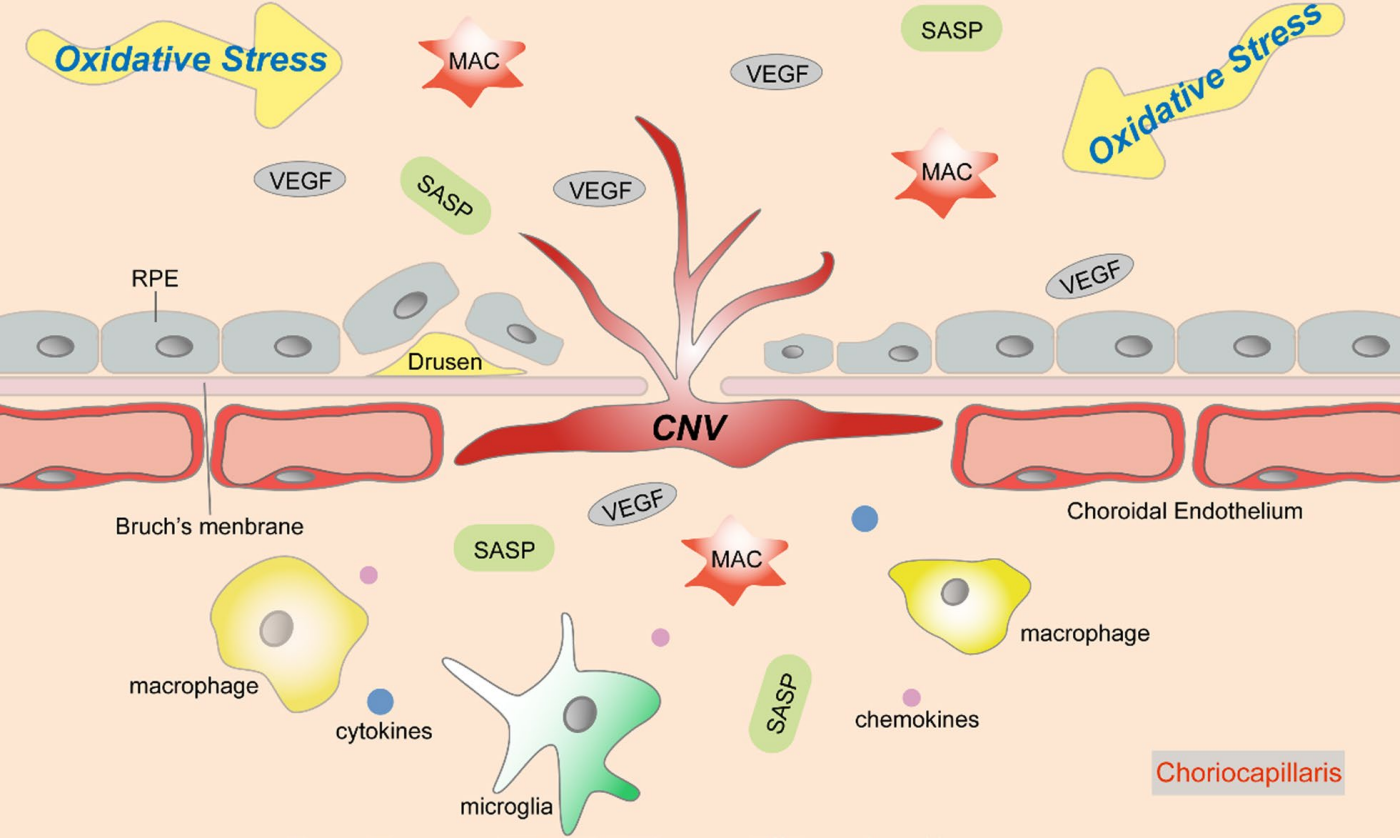
The role of oxidative stress in Choroid-retinal pigment epithelium (RPE) damage during the progression of age-related macular degeneration (AMD). Oxidative stress induces ischemia and chronic inflammation in Choroid-RPE complex. Excess production of VEGF in response to ischemia initiates pathological angiogenesis, and then choroidal neovascularization (CNV) develops as a consequence. In addition, oxidative stress contributes to overactivation of inflammatory responses, consisted of the accumulation of membrane attack complex (MAC) and senescence-associated secretory phenotype (SASP), as well as the recruitment of activated macrophages and microglia

In response to oxidative stress, overactivated angiogenetic signals and immune responses synergistically exert detrimental effects on choriocapillaris during AMD progression (Fig. 2).

## Epigenetic regulation of ocular vascular aging

**Fig. 3:**
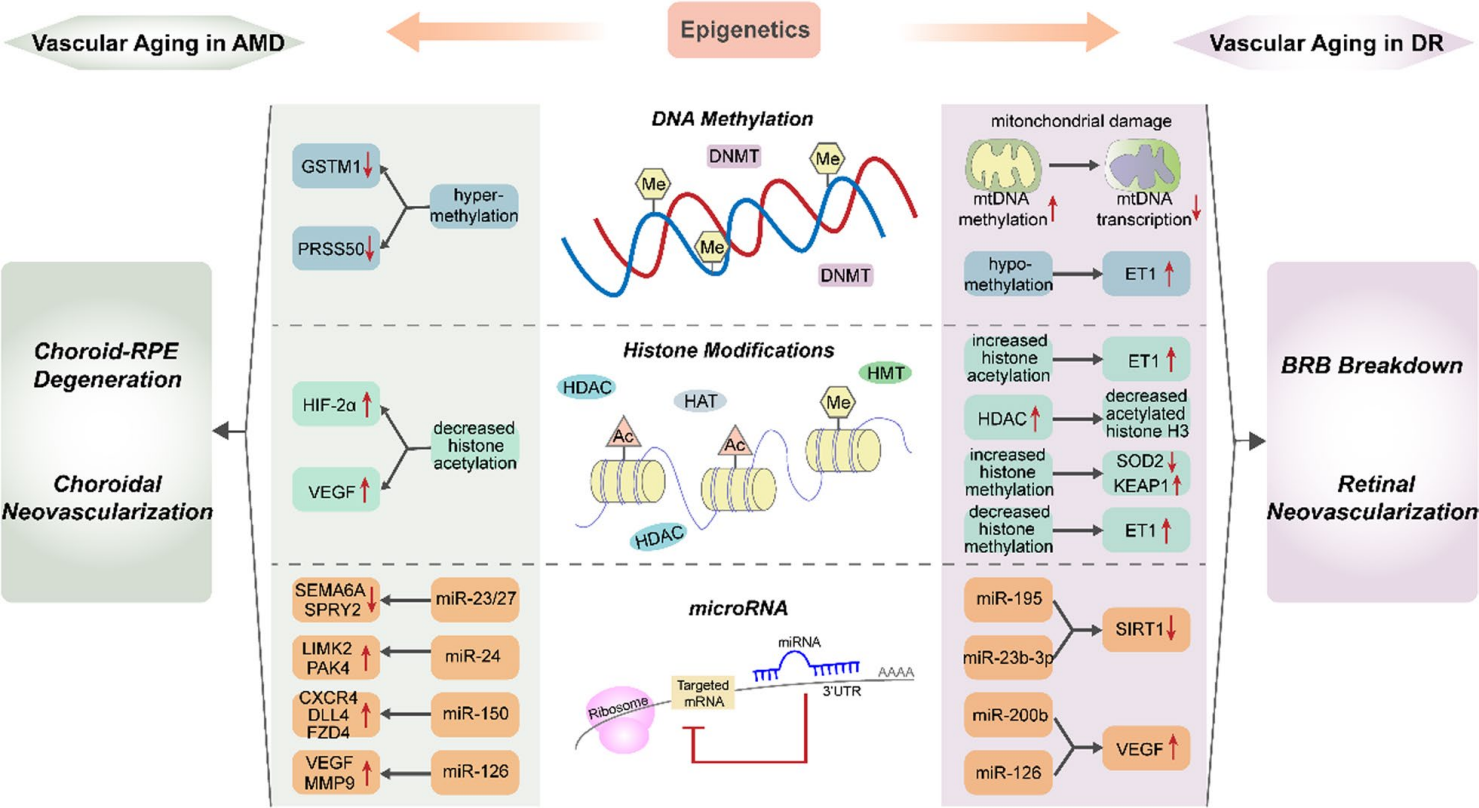
Diagram of the epigenetic regulation of vascular aging in DR and AMD. The dysregulation of DNA methylation, histone modifications and microRNA in microvessels induces alterations in the expression of targeted genes, which are responsible for vascular aging in the process of DR and AMD. AMD: age-related macular degeneration, CXCR4: C-X-C chemokine receptor type 4, DLL4: Delta like ligand 4, DNMT: DNA methyltransferase, DR: diabetic retinopathy, ET1: endothelin-1, FZD4: frizzled-4, GSTM1: glutathione S-transferase isoform mu1, HAT: histone acetyltransferase, HDAC: histone deacetylase, HIF-2α: hypoxia inducible factor 2α, HMT: histone methyltransferase, KEAP1: Kelch-like ECH-associated protein 1, LIMK2: LIM domain-containing kinase 2, MMP9: matrix metalloproteinase 9, mtDNA: mitochondrial DNA, PAK4: P21-activated kinase 4, PRSS50: protease serine 50, SEMA6A: semaphorin6A, SIRT1: sirtuin 1, SOD2: superoxide dismutase 2, SPRY2: sprouty RTK signaling antagonist 2, VEGF: vascular endothelial growth factor

Epigenetic modification induces long-term alterations in gene expression, which acts as a linkage between the genetic inheritance and the environment in the process of aging and diseases (Kowluru et al. 2015). Epigenetic regulation consisted of DNA methylation, histone modifications, non-coding RNAs and chromatin remodeling, is intrinsically implicated in retinal vascular diseases (Mohana Devi et al. 2021). As stated above, vascular aging plays a key role in the onset and progression of DR and AMD. Therefore, we focus on the interaction between epigenetics and vascular aging underlying the pathogenesis of these diseases (Fig. 3).

### The role of epigenetics in vascular aging of DR

Retinal microvessels from DR donors exhibited an increase in methylation modification of mitochondrial DNA (mtDNA) and a decrease in mtDNA transcription, compared with the vessels from age-matched non-DR donors. Hyperglycemia upregulated the expression of DNA methyltransferase DNMT1 and mtDNA methylation in human retinal endothelial cells, leading to mitochondrial damage as well as cell apoptosis (Mishra and Kowluru 2015). In DR rats, elevated methylation levels of mtDNA contributed to decreased mitochondrial copy numbers and retinal acellular capillaries, which suggested the association of DNA methylation dysregulation with vascular impairments. Interestingly, the investigators found aggravated ROS burden and mtDNA methylation in Type 2 diabetes compared with Type 1 diabetes rats, which indicated that obesity accelerated the hyperglycemia-induced oxidative impairments and epigenetic modification in retinal vasculature (Kowluru 2020). Upregulation of endothelin-1 (ET-1) is correlated with vascular dysfunction in diabetic complications (Ergul 2011). To elucidate the mechanism of aberrant expression of ET-1 in hyperglycemia, retinal vascular endothelial cells were treated with HG followed by epigenetic analyses of DNA methylation, histone methylation, and long non-coding RNA (lncRNA)-mediated regulation of ET-1 expression. Hypomethylation of CpG sites within its promotor was detected in response to HG treatment. Inhibition of DNA or histone methylation facilitated ET-1 transcription in endothelial cells. In addition, interfering the pathogenetic lncRNA ANRIL, MALAT1, or ZFAS1 protected the retinal endothelial cells against glucose-induced ET-1 upregulation (Biswas et al. 2018). Another group uncovered histone acetylation-mediated regulatory mechanism of ET-1 expression. Human microvascular endothelial cells exposed to HG showed upregulation of ET-1 and decreased expression and activity of Sirt1, a class III histone deacetylase (HDAC). Sirt1 overexpression reversed malfunction of histone acetylation and ET-1 transcription, by which endothelium was preserved from HG-induced hyperpermeability (Mortuza et al. 2015). Furthermore, there are studies showing the correlation of deficient histone acetylation with vascular aging. Retinas of diabetic rats exhibited a decrease in acetylated histone H3 levels along with overactivation of HDAC. Hyperglycemia improved HDAC expression in retinal endothelium with the effect of decayed histone acetylation (Zhong and Kowluru 2010). miRNAs are small non-coding RNAs that regulate gene expression at the post-transcriptional level. In recent decades, growing studies have illuminated the involvement of miRNAs dysregulation in diabetic vasculopathy (Mastropasqua et al. 2014). miRNA RT-PCR array identified 120 miRNAs with differential expression in primary retinal endothelial cells isolated from diabetic rats compared to the controls (Kovacs et al. 2011). Therein, NF-κB responsive miRNAs miR-21, miR-132, miR-146a, miR-146b, and miR-155 were verified to be upregulated in diabetic endothelial cells, which indicated that miRNAs participated in DR microvascular injury via regulating NF-κB-mediated endothelial inflammatory responses (Kovacs et al. 2011). HG treatment augmented miR-195 expression in human retinal endothelial cells accompanied by decreased Sirt1 transcription. miR-195 exerted inhibitory effect on Sirt1 expression by binding to Sirt1 3’UTR. Accordingly, miR-195 antagomir restored endothelial Sirt1 expression and antioxidative defense, and as a consequence counteracted endothelial senescence induced by hyperglycemia (Mortuza et al. 2014). miR-23b-3p also serves as an executioner to vascular dysfunction in DR through negatively regulating Sirt1 expression in endothelial cells (Zhao et al. 2016). In proliferative DR, retinal NV can be partially attributed to excessive production of VEGF. Increased VEGF level was detected in bovine retinal endothelial cells exposed to HG, in which miR-200b abundance was significantly suppressed. Transfection of miR-200b mimic normalized endothelial VEGF expression, maintained endothelial permeability and prevented inducible angiogenesis. In human retinal sections, miR-200b was expressed in nondiabetic capillaries, however DR impaired the endothelial localization of miR-200b, which supported the results of the cellular experiments (McArthur et al. 2011). Furthermore, it was found that in a retinal ischemia model mimicking DR, recovering miR-126 expression prohibited the overproduction of VEGF, and ameliorated retinal pathological NV (Bai et al. 2011). As sponges of miRNAs, another type of non-coding RNAs, circular RNAs can modulate the degeneration of endothelial cells and pericytes in diabetic vascular dysfunction through interfering miRNAs activity (Shan et al. 2017; Jiang et al. 2020).

### The effect of epigenetic regulation on vascular aging in AMD

AMD patients showed hypermethylation of glutathione S-transferase isoform mu1 in correspondence to its decreased expression in the Choroid-RPE samples compared with age-matched controls (Hunter et al. 2012). An elevation of DNA methylation was also detected within the promotor region of protease serine 50 in both blood and retina samples with AMD (Oliver et al. 2015). To date, there is no evidence demonstrating the direct correlation of DNA methylation in choriocapillaris or component cells with AMD pathology. Choroidal neovascular membranes excised from AMD patients showed strong immunoreactivity for hypoxia inducible factor 2α (HIF-2α) and Sirt1, which suggested the role of histone deacetylation in the formation of CNV (Sheridan et al. 2009; Maloney et al. 2013). Sirt1 inhibition reversed the hypoxia-mediated elevation of HIF-2α and VEGF in choroidal endothelial cells (Balaiya et al. 2012). In a laser-induced CNV model, increased histone acetylation via administration of a HDAC inhibitor mitigated CNV invasion and vascular leakage from laser lesions (Dahbash et al. 2019). Coincidently, HDAC suppression disturbed angiogenesis of choroidal endothelial cells in response to VEGF, and attenuated CNV formation and leakage in a mouse model simulating neovascular AMD (Chan et al. 2015). Mounting studies have highlighted that CNV lesions could be relieved by manipulating miRNAs. miR-23/27 expression was positively associated with angiogenesis, therefore anti-miR-23/27 treatment had beneficial effect on repressing CNV (Zhou et al. 2011). miR-24 targeted Rho signaling pathway to regulate actin cytoskeleton dynamics in endothelium, which was essential for endothelial migration and angiogenesis. Subretinal delivery of miR-24 mimics attenuated laser-induced CNV in an experimental model for AMD (Zhou et al. 2014). In addition, miR-150 knockout mice showed aggravated CNV lesions after photocoagulation, which demonstrated the inverse correlation between miR-150 expression and choroidal vascular impairments (Liu et al. 2015). Surprisingly, miR-126-3p possessed dual function in CNV formation dependent on targeted cell types. Referring to endothelial cells, miR-126-3p silencing repressed pathological angiogenesis, which was supported by compromised CNV area in a mouse CNV model following anti-miR-126-3p treatment. In exudative AMD, VEGF arisen from ischemia triggered the formation of CNV. miR-126-3p mimic inhibited VEGF secretion from RPEs, and downregulated total VEGF levels in the laser-injured retina, which was responsible for impedance of CNV (Zhou et al. 2014). Another group reported the negative regulatory role of miR-126 in hypoxia-induced VEGF production in choroidal endothelial cells (Ye et al. 2014). Furthermore, miR-126 manipulated endothelial inflammatory responses through regulating the adhesion molecules and cytokines (Harris et al. 2008; Liu et al. 2021). Overall, the bidirectional effects of miR-126 on vascular aging in AMD was contributed from the multiple regulatory mechanisms of miR-126 in Choroid-RPE complex.

## The interplay between oxidative stress and epigenetics in retinal vascular aging

There are complicated interactions between oxidative stress and epigenetic modifications in diabetic retinal vascular aging. Retinas of diabetic mice showed decreased expression of the manganese superoxide dismutase SOD2, in coincidence with an increment of trimethyl histone H4 lysine 20 (H4K20me3) at SOD2 promotor. Moreover, the upregulation of SUV420h2, one of the prime enzymes for the trimethylation of histone H4K20, was detected in diabetic retinas. To explore whether histone methylation was correlated with antioxidant defects in the process of vascular aging in DR, SUV420h2 knockdown was performed in retinal endothelial cells followed by HG treatment. Deprivation of SUV420h2 normalized SOD expression through inhibiting histone methylation, which indicated the epigenetic regulation of endothelial antioxidative defense (Zhong and Kowluru 2011). In the retinas of human donors with DR, the expression of Kelch-like ECH-associated protein 1 (Keap1) was elevated accompanied with the enhanced binding of transcriptional factor Sp1 at Keap1 promotor (Mishra et al. 2014). Keap1 is identified as a negative regulator of Nrf2 activity through tethering Nrf2 in the cytosol and inhibiting Nrf2 nuclear transport (Lu et al. 2016). Diabetes enhanced Keap1-Nrf2 interactions, and decreased the nuclear accumulation of Nrf2, by which the transcription of antioxidants was suppressed (Zhong et al. 2013). In retinal endothelial cells, HG treatment improved Sp1 recruitment at Keap1 promotor and then enhanced Keap1 expression. As a consequence, the activities of Nrf2 and its downstream genes were depressed, which indicated the malfunction of antioxidative defense. Furthermore, the enrichment of methylated histone H3K4 at Keap1 promotor was detected in endothelial cells challenged with HG, in coincidence with increased histone methyltransferase SetD7. Inhibition of SetD7 expression reversed the upregulation of Keap1 via modulating histone methylation, and consequently rescued the Nrf2-mediated transcription of antioxidants (Mishra et al. 2014). In conclusion, the epigenetic modification of Keap1 regulated endothelial oxidative response via Keap1-Nrf2 interaction.

## Perspective and future directions

To date, clinical pharmacological treatment for DR and AMD mainly focuses on anti-VEGF therapies, which targets at the inhibition of NV at advanced stage of the diseases. However, not all patients respond optimally, and there are potential adverse effects arisen from frequent intravitreal injections, such as the risk of endophthalmitis and retinal detachment (Gahlaut et al. 2015). Considering the key role of vascular aging in the onset and progression of the diseases, inhibition of vascular aging is reasonable to be an alternative therapeutic strategy for DR and AMD, especially at the early stage. In this review, we demonstrate the involvement of oxidative stress and epigenetics in the ocular vascular aging. Protecting vascular cells against aging insults contributes to improved outcomes in experimental DR or AMD models. Further investigation could proceed with the clinically applicable interventions of vascular aging for treatments for age-related vascular diseases.

## Data Availability

All data generated or analysed during this study are included in this published article.

## Abbreviations

AMD: Age-related macular degeneration
BRB: Blood-retinal barrier
CCL2: Chemokine (C-C motif) ligand 2
CFH: Complement factor H
CNV: Choroidal neovascularization
DME: Diabetic macular edema
DR: Diabetic retinopathy
ET-1: Endothelin-1
H4K20: Histone H4 lysine 20
HDAC: Histone deacetylase
HG: High glucose
HIF-2α: Hypoxia inducible factor 2α
Keap1: Kelch-like ECH-associated protein 1
lncRNA: Long non-coding RNA
MAC: Membrane attack complex
mtDNA: Mitochondrial DNA
NF-κB: Nuclear factor-κB
NO: Nitric oxide
Nrf2: Nuclear factor-erythroid 2-related factor-2
NV: Neovascularization
RNS: Reactive nitrogen species
ROS: Reactive oxygen species
RPE: Retinal pigment epithelium
SASP: Senescence-associated secretory phenotype
Sirt1: Sirtuin 1
SOD: Superoxide dismutase
VEGF: Vascular endothelial growth factor
